# Adherence to COVID-19 preventive measures among residents in selected townships, Yangon Region, Myanmar: a community-based cross-sectional study

**DOI:** 10.1186/s41182-024-00603-6

**Published:** 2024-05-11

**Authors:** Ye Minn Htun, Nyan Lin Maung, Dwe Kyaw Ko, Han Myo Htut, Min Khant Phyo, Wai Lynn Aung, Hein Khant Zaw, Aung Kyaw Min, Aung Phyo Kyaw, Thet Swe, Kaung Khant Zaw, Kyaw Swar Naing Win, Khant Ko Ko, Khant Min Thaw, Saw Pyae Aung, Saw Yan Aung, Soe San Htun, Soe Htet Paing, Soe Lin Htun, Zaw Myo Naing, Zin Ko Htun, Htoo Naung, Htun Htun Oo, Naing Ye Hla, Aung Kyaw San, Hpone Myint Myat, Phone Shan Htet, Min Khant Mon, Ye Myat Paing, Wai Lin Phyo, Win Khant Paing, Thu Rein, Thit Lwin Oo, Thet Paing Zaw, Thet Lynn Oo, Thint Myat Thu, Than Toe Aung, Hein Htet Soe, Aung Kyaw Soe, Aung Myint Oo, Aung Aung, Pyae Phyo Aung, Htun Aung Kyaw, Hpone Pji Kyaw, Yan Naing Myint Soe, Myint Myat Ko, Zin Ko Aung, Kyaw Thiha Aung, Yan Paing Chit Lwin, Wai Yan, Phyo Tayza Soe, Zin Linn Htet, Nay Hein Sint, Zayar Aung, Zin Thu Winn, Kaung Si Thu, Nyan Htet Shan, Nyan Sint Htun, Tun Tun Win, Kyaw Myo Tun

**Affiliations:** 1 Department of Prevention and Research Development of Hepatitis, AIDS and Other Viral Diseases, Health and Disease Control Unit, Nay Pyi Taw, 15011 Myanmar; 2 Department of Research and Development, Defence Services Medical School, Yangon, Myanmar; 3 Department of Preventive and Social Medicine, Defence Services Medical Academy, Yangon, Myanmar; 4 Outpatient Department, No. 1 Military Hospital (500 Bedded), Meiktila, Mandalay Myanmar

**Keywords:** Adherence, COVID-19, Pandemic, Preventive measures, Residents, Myanmar

## Abstract

**Background:**

To fight the current coronavirus disease (COVID-19) pandemic, many countries have implemented various mitigation measures to contain the spread of the disease. By engaging with health service providers, the community’s participation in adherence to preventive measures is certainly required in the implementation of COVID-19 mitigation strategies. Therefore, this study aimed to assess the level of adherence to COVID-19 preventive measures and its associated factors among the residents, Yangon Region, Myanmar.

**Methods:**

A community-based cross-sectional study was carried out among 636 residents in Yangon Region, Myanmar, from October to December 2021. A multistage non-probability sampling method, purposively selected for three townships in Yangon Region and convenience sampling for 212 participants from each township, was applied and the data were collected by face-to-face interviews using structured and pretested questionnaires. Data were entered, coded, and analyzed using IBM SPSS version 25.0. Simple and multiple logistic regression analysis were performed to identify the significant variables of adherence to COVID-19 preventive measures.

**Results:**

As a level of adherence to COVID-19 preventive measures, the proportion of residents who had good adherence was 39.3% (95% CI 35.5–43.2%), moderate adherence was 37.6% (95% CI 33.8–41.5%), and poor adherence was 23.1% (95% CI 19.9–26.6%). The age group of 31–40 years (AOR: 3.13, 95% CI 1.62–6.05), 30 years and younger (AOR: 3.22, 95% CI 1.75–5.92), Burmese ethnicity (AOR: 2.52, 95% CI 1.44–4.39), own business (AOR: 3.19, 95% CI 1.15–8.87), high school education level and below (AOR: 1.64, 95% CI 1.02–2.69), less than 280.90 USD of monthly family income (AOR: 1.51, 95% CI 1.01–2.29), low knowledge about COVID-19 (AOR: 1.90, 95% CI 1.26–2.88) were significantly associated with poor adherence to COVID-19 preventive measures.

**Conclusions:**

In this study, nearly one-fourth of the residents were experiencing poor adherence to COVID-19 preventive measures. Therefore, building up the risk communication through the community using widely used mainstream media, the continuation of disease surveillance and announcement of updated information or advice for the public to increase awareness towards COVID-19, and enforcement to follow the recommended directions and regulations of health institutions are vital to consider for improving the adherence to preventive measures against COVID-19 among the residents.

**Supplementary Information:**

The online version contains supplementary material available at 10.1186/s41182-024-00603-6.

## Background

Severe acute respiratory syndrome coronavirus 2 (SARS-CoV-2), which leads to the contagious disease, coronavirus disease 2019 (COVID-19), was first reported in December 2019 in Wuhan, China and has become a pandemic without precedent after spreading quickly over the world [1, 2]. The virus spreads mainly between people who are in close contact with each other, through airborne transmission, and droplet transmission [3]. SARS-CoV-2 changes over time and some changes may affect the virus’s properties, such as increased transmissibility, disease severity, the performance of vaccines, therapeutic medicines, diagnostic tools, or public health and social measures [4]. To keep the COVID-19 pandemic under public health control, many countries around the world have implemented several preventive measures with various strategies such as complete or partial lockdowns, travel ban, improving testing capacity, contact tracing, maintaining social distancing, keeping physical distance, quarantine, frequent hand washing, covering coughs or sneezes, and avoiding contamination of face with unwashed hands [5–10].

In Myanmar, on 4th January 2020, the Ministry of Health and Sports (MOHS), former name of Ministry of Health (MOH), was notified by the WHO Regional Office for South East Asia and ASEAN + 3 Senior Officials’ Meeting on Health Development about unexplained pneumonia cases in Wuhan City, China. Then, the government started the preparedness mainly in points of entry and commenced the risk communication to the population, on 5th January 2020 [11]. On 13th March 2020, for the COVID-19 response, the State Counsellor established the National Level Central Committee on Prevention, Control, and Treatment of COVID-19 and formed the working committee to address the possible impacts of COVID-19 on the country’s economy. The first COVID-19 laboratory-confirmed cases were reported on 23rd March 2020, and then, the disease spread across the country. In the first wave of COVID-19 epidemic, there were 0.73 confirmed cases per 100,000 population with a 1.60% case fatality rate (CFR) [12, 13]. The government of Myanmar established several restrictions including 14-day quarantine for all incoming travelers, an entry ban for all countries, the suspension of international commercial flights, bans on public gatherings, closures of public events, entertainment venues, and religious institutions, and lockdown and stay-at-home at the high-risk townships [11, 14].

In mid-August 2020, the second wave started from Rakhine State and there were 278 confirmed cases per 100,000 population with 2.25% CFR during this wave [11, 12, 14, 15]. To reduce the community spread of COVID-19, the government performed preventive measures such as a strengthening of testing capacity at fever clinics and hospitals, expansion of designated quarantine and treatment centers, lockdown and stay-at-home restrictions at the high-risk townships, restriction on public gathering and movement, closure of restaurants and child care facilities, deferral of international flights, and enforcement of contact tracing. In January 2021, Myanmar launched a Myanmar National COVID-19 Vaccine Deployment Plan that was started with priority groups, such as healthcare workers and frontline health volunteers [11, 15].

At the end of May 2021, a third wave of COVID-19 hit the whole country with the highest impact on lives and the economy. There were 760 confirmed cases per 100,000 population with 4.11% CFR in this wave. To reduce the community spread, MOH performed the mask campaign and risk communication, quarantine and screening to all travelers and returnees, scaling up the testing and treatment facilities, school and office closures, and stay-at-home restrictions in risky areas [13, 16].

From 28th January 2022, the COVID-19 confirmed cases surged again and then the fourth wave of COVID-19 was started in Myanmar. MOH is taking public announcements of daily confirmed cases and deaths, risk communication messages, and advice for the public regarding prevention and control measures through the official website and social media. Strict quarantine and COVID-19 testing procedures were being performed for all travelers at the points of entry. MOH is also implementing the mitigation measures such as the expansion of testing capacity in both public and private sectors, providing treatment at designated treatment centers, and enforcing to get vaccination at least 70% of the population. As of 30th April 2023, the MOH reported 634,877 confirmed cases of COVID-19, with 19,492 deaths or a CFR of 3.07% [12, 13].

Aiming to reduce disease transmission, morbidity, and mortality, several countries have implemented a series of non-pharmaceutical interventions as COVID-19 preventive measures regarding protective behaviours, such as wearing masks, keeping a physical distance, covering coughs and sneezes, handwashing, and avoiding social gatherings [17]. The public knowledge and attitudes towards COVID-19 play an integral role in performing the preventive measures recommended by the health authorities and also attribute to adhere these measures even if the COVID-19 confirmed cases decreased [18, 19]. It is critical to promote adherence to preventive measures by all means so as to tackle and mitigate the spread of SARS-CoV-2. Nevertheless, the ongoing noncompliance of a subgroup of the population to the COVID-19 preventive measures can be a major challenge [20–24]. Assessing the level of adherence to preventive measures related to COVID-19 among the population would be helpful to provide better insight to address which preventive measures would be implemented to match transmission dynamics and evaluate the development of preventive strategies and health promotion programs. A socio-behavioural study stated that there were 87% of low knowledge about COVID-19 and only 22% of reported good protective behaviours [25]. However, to date, there is inadequate information on the community’s adherence to COVID-19 preventive measures in Myanmar. Therefore, this study aimed to assess the level of adherence to COVID-19 preventive measures and its associated factors among residents in Myanmar.

## Methods

### Study design, area, and population

**Fig. 1:**
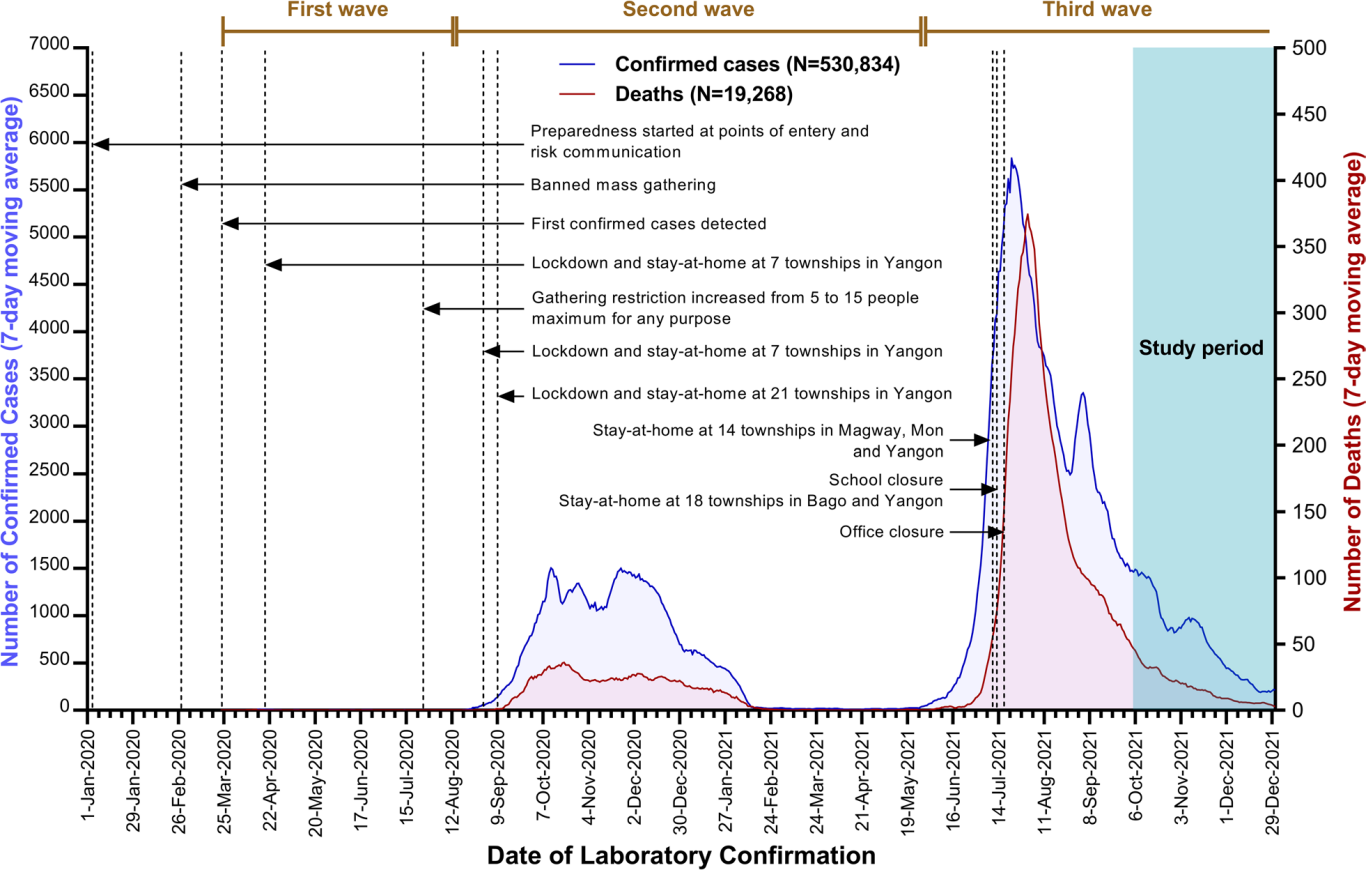
Epidemic curve of COVID-19 confirmed cases and deaths with the main containment measures in Yangon Region and the whole country, as of 31st December 2021 (Data source: https://mohs.gov.mm/Main/content/publication/2019-ncov)

A community-based cross-sectional study was conducted in Yangon Region, Myanmar, from October to December 2021, beyond the peak of the third wave. Myanmar had reported 530,834 COVID-19 confirmed cases with 19,268 deaths, 16.5 million people who have been fully vaccinated, and 4.4 million people administered the first dose of the COVID-19 vaccine, as of 31st December 2021. The epidemic curve of COVID-19 confirmed cases and deaths with the main containment measures in Yangon Region and the whole country, as of 31st December 2021 is shown in Fig. 1. The study area, Yangon Region, sits within the wider Delta Region of the south, sharing borders with Ayeyarwady Region to the west, and Bago Region to the north and east, and resting on the Andaman Sea to the south. Yangon Region covers a span of 10,171 km^2^ administratively divided into 46 townships. It is a highest population density area with an estimated population of 7.3 million and a population density of 716.3 people per square kilometer. Seventy percent of the population lives in urban areas, and the sex ratio is 92 males per 100 females [26]. The majority of COVID-19 confirmed cases were reported from the Yangon Region, which became an epicenter of all epidemic waves in Myanmar.

### Sample size determination and sampling procedure

**Fig. 2:**
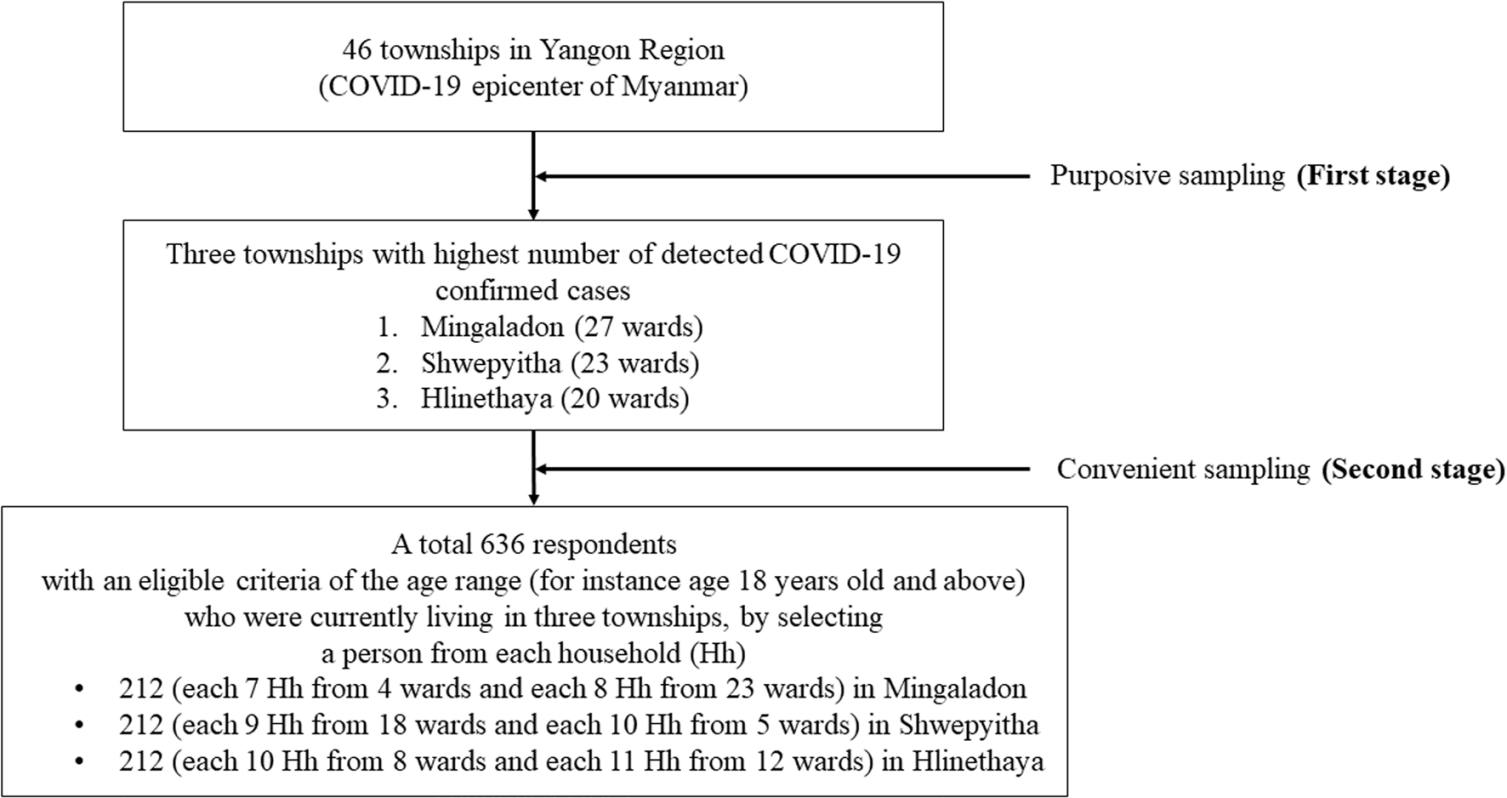
Flow chart showing sampling procedure

The sample size was determined using single population proportion formula [27], with an assumption of a 4% margin of error (d = 0.04), 95% confidence interval (α = 0.05), and 59.3% of poor adherence to COVID-19 preventive measures (*p* = 0.59) [28]. To anticipate the problem of non-response and avoid underestimation of sample size, there were 10% recruiting more participants on top of the minimum sample size [29] and then the final minimum required sample size for this study was 636. To select the participants, a multistage non-probability sampling technique was applied, supposing the highly importance of adherence to preventive measures among the population living in highest case detected areas. Firstly, as stated by the COVID-19 data from MOH, three townships with the highest number of detected COVID-19 confirmed cases (Mingaladon, Shwepyitha, and Hlinethaya) were purposively selected from the 46 townships of the Yangon Region. Then, 212 participants from each township were collected by convenient sampling technique. For the recruitment of participants, all adult residents who were currently living in Mingaladon, Shwepyitha, and Hlinethaya townships were eligible for this study. The research team selected an eligible respondent from each household based on the geographical proximity. Flow chart for sampling procedure is shown in Fig. 2.

### Data collection tool and procedures

The data were collected by the pretested structured questionnaires that were designed for the purpose of the study and adapted based on the previous literature conducted among the general population for the assessment of adherence to COVID-19 preventive measures [20, 30–33]. The questionnaires consisted of five parts: sociodemographic characteristics, COVID-19 epidemic-related factors, knowledge about COVID-19, attitudes towards COVID-19, and adherence to COVID-19 preventive measures. The first part was constructed for the sociodemographic characteristics, including sex, age, marital status, ethnicity, religion, occupation, education, living situation, household members, monthly family income, and comorbidity. The second part consisted of the factors related to the COVID-19 epidemic such as sources of information about COVID-19, being infected with COVID-19, family members infected with COVID-19, and who received the COVID-19 vaccine. In the third part, there were 12 items for the knowledge about COVID-19. The items 1, 2, 5, 6, 8, 9, 10, and 11 allowed the option of “yes”, “no”, and “don’t know” while others (items 3, 4, 7, and 12) permitted multiple responses (Additional file 1). The scoring for the knowledge questions was one point for a correct answer, and zero point for an incorrect answer or a “don’t know” response. The total score of the knowledge questions ranged from 0 to 35.

In the fourth part, the level of attitudes towards COVID-19 was assessed by ten statements including positive and negative aspects. A five-point Likert’s scale was used to assess the level of agreement with the statements: strongly agree (five points), agree (four points), uncertain (three points), disagree (two points), and strongly disagree (one point) for the positive statement (item 1, 2, 3, 4, 5, 7, and 9) and reverse scoring for the negative statements (item 6, 8 and 10). The scores for the attitudes towards COVID-19 ranged from 10 to 50. The fifth part comprised 14 items to assess the adherence to COVID-19 preventive measures and a five-point Likert’s scale was used for the scoring of the statements: always (five points), often (four points), sometimes (three points), rarely (two points), and never (one point). The scores for adherence to COVID-19 preventive measures was a range of 14–70.

The questionnaires were initially prepared in English and then translated into the Burmese language, the local language. The pretest was done on 60 residents (10% of the total required sample size) in Insein township, one of the townships with highly detected COVID-19 confirmed cases, to assess the reliability. Based on the results of the pretest, modifications have been applied to adjust the items of the questionnaires but the pretested data were not included in the final analysis. The reliability of the questionnaires was assessed by calculating the Cronbach’s α coefficients which were found to be satisfactory for the two parts of the questionnaires (0.74 for attitudes towards COVID-19 and 0.79 for the adherence to preventive measures for COVID-19). The questionnaires in the Burmese language were used for the data collection after refining based on pretested results (Additional file 2).

### Operational definitions

*Comorbidity* defined as the residents who had a medical record of having one or more chronic diseases such as hypertension, diabetes mellitus, cardiovascular diseases, cancer, chronic kidney disease, chronic liver disease, chronic lung disease, and people with living HIV.

*Got COVID-19 vaccine* defined as the residents who were fully vaccinated or got first doses of COVID-19 vaccine.

*Knowledge about COVID-19* defined as the concept of the residents regarding causes, routes of transmission, symptoms, the severity of COVID-19, and understanding of COVID-19 preventive measures. Of 35 total scores of knowledge questions, the level of knowledge was categorized using Bloom’s cutoff point: less than 60% scores for “low”, 60–79% scores for “moderate”, and 80–100% scores for “high” [34–36].

*Attitudes towards COVID-19* defined as the thinking or feeling of the residents regarding COVID-19 and based on the total scores of 50, the level of attitudes was categorized using Bloom’s cutoff point: less than 60% scores for “negative”, 60–79% scores for “neutral”, and 80–100% scores for “positive” [34–36].

*Adherence to COVID-19 preventive measures* defined as the residents’ practices or compliance with COVID-19 prevention measures, endorsed by the government such as hand washing, using a facemask, keeping physical distance, covering nose and mouth with bent elbow or tissue when coughing or sneezing, not traveling to a crowded place, homestay, and keeping away from the unprotected direct contact with live animals, regularly practiced during 14 days before data collection time. On the total score of 70, the level of adherence was categorized using Bloom’s cutoff point: less than 60% scores for “poor”, 60–79% scores for “moderate”, and 80–100% scores for “good” [34–36].

### Data quality control

There were three data collection teams (for three townships) in this study and 15 data collectors were involved in each team, who were supervised by three senior public health physicians. A two-day training was provided to the data collectors for the objective of the study, data collection procedures, COVID-19 precautions, and ethical considerations. Appropriate or pertinent instructions and directions were given for the data collectors to ensure the quality of the data. The collected data were daily checked for completeness and consistency by the principal investigator and supervisors. Any confusion on the data collection procedure and responses were handled in a timely manner.

### Statistical analysis

After checking data completeness and consistency, the collected data were coded and entered into Microsoft Excel 2016 (Additional file 3). Then the data were transferred to the IBM SPSS Statistics for Windows, Version 25.0 (Armonk, NY: IBM Corp) for cleaning and analysis. Descriptive statistics were used to generate frequency tables, expressing the number with percentage for the categorical variables and mean with standard deviation (± SD) or median with interquartile range (IQR) for the continuous variables. For the final analysis, binary logistic regression was chosen in order to predict the probability of the outcome variable given the predictor variables, identify the strength of association, and avoid the confounding effects [35, 37]. Therefore, the level of knowledge was expressed as “low knowledge-yes” (low level of knowledge) and “low knowledge-no” (combined moderate and high level of knowledge), the level of attitudes was expressed as “negative attitudes-yes” (negative level of attitudes) and “negative attitudes-no” (combined neutral and positive level of attitudes), and the level of adherence was expressed as “poor adherence-yes” (poor level of adherence) and “poor adherence-no” (combined moderate and good level of adherence). After checking the model fitness and fulfillment of the assumption by the Hosmer–Lemeshow goodness of fit test, binary logistic regression analysis was performed to find out the associated factors of adherence to COVID-19 preventive measures. All significant independent variables in bivariate analysis (age, ethnicity, occupation, education, monthly family income, comorbidity, infected with COVID-19, low knowledge, and negative attitudes) were considered in the multiple logistic regression analysis. The strength of association was described as crude odds ratio (COR) and adjusted odds ratio (AOR) with a 95% confidence interval (CI). The independent variables with a *p* value ≤ 0.05 were considered statistically significant.

## Results

There were 636 residents who gave informed consent and participate in this study. The sociodemographic characteristics of the participants are shown in Table 1. Among the total, 47.2% were male residents and 52.8% were female. The mean (± SD) age of the participants was 33.94 (± 12.37) years with a range of 18–70 years and nearly half of the participants, 49.1%, were 30 years and younger. Of all participants, 55.3% were married, 78.6% were Burmese ethnicity, and 86.0% were Buddhists. For the occupation, 31.9% were private employees and 68.1% were the other categories: dependants 25.2%, own business 22.3%, government staff 10.5%, and unskilled laborers 10.1%. Regarding education, 33.0% of participants passed the high school education, and 20.4% were graduates and above. In the living situation, 89.0% of participants were living with their families and only 4.1% were living alone. The median family member (IQR) was 4 (2, 3–5) with a minimum of 1 to a maximum of 10, and 53.1% of participants were living with four household members and more. The median (IQR) monthly family income was 280.90 (146.07: 191.01–337.08) USD with a range of 78.65–1123.60 USD and 51.3% of participants earned 280.90 USD and more monthly family income. Among the total, 13.8% of participants had comorbidity.

**Table 1:** Sociodemographic characteristics of the participants

Variables	*n *(%)
Sex	
Male	300 (47.2)
Female	336 (52.8)
Age (year)	
≤ 30	312 (49.1)
31–40	170 (26.7)
> 40	154 (24.2)
	Mean (± SD): 33.94 ± 12.37 years, Minimum 18 years, Maximum 70 years
Marital status	
Single	230 (36.2)
Married	352 (55.3)
Separate	14 (2.2)
Divorced	12 (1.9)
Widowed	28 (4.4)
Ethnicity	
Burmese	500 (78.6)
Rakhine	51 (8.0)
Kayin	48 (7.5)
Chin	28 (4.4)
Mon	6 (0.9)
Shan	3 (0.5)
Religion	
Buddhist	547 (86.0)
Christian	86 (13.5)
Hindus	3 (0.5)
Occupation	
Dependant	160 (25.2)
Unskilled laborer	64 (10.1)
Own business	142 (22.3)
Private employee	203 (31.9)
Government staff	67 (10.5)
Education	
Illiterate	9 (1.4)
Read and write	31 (4.9)
Primary school education	18 (2.8)
Middle school education	124 (19.5)
High school education	210 (33.0)
College and university	114 (17.9)
Graduate and above	130 (20.4)
Living situation	
Alone	26 (4.1)
With family	566 (89.0)
With friend(s)	44 (6.9)
Household member	
< 4	298 (46.9)
≥ 4	338 (53.1)
	Median (IQR): 4 (2: 3–5), minimum 1, Maximum 10
Monthly family income (USD) *	
< 280.90	310 (48.7)
≥ 280.90	326 (51.3)
	Median (IQR): 280.90 (146.07: 191.01–337.08) USD, Minimum 78.65 USD, Maximum 1123.60 USD
Comorbidity	
Present	88 (13.8)
Absent	548 (86.2)

*Monthly family income was expressed by USD according to the reference exchange rate (1 USD = 1780 Kyat) of Central Bank of Myanmar during the study period. Source: https://forex.cbm.gov.mm/index.php/fxrate (Median (IQR): 500,000 (260,000: 340,000–600,000) Kyat, Minimum 140,000 Kyat, Maximum 2,000,000 Kyat)

The COVID-19 epidemic-related factors of the participants are expressed in Table 2. As the main sources of information, 48.7% of participants accessed information about COVID-19 from the government media (MOH website and Facebook page, Myanmar Radio and Television broadcasting), 36.2% from health workers, and 26.6% from social media. The rest, 15.4% and 11.9%, received the COVID-19 information from their friends and family members, respectively. Among the total, 40.6% of the participants infected with COVID-19, 47.6% responded that their family members were infected with COVID-19, and 63.4% got the COVID-19 vaccine. As shown in Table 3, 37.7% (95% CI 34.0–41.6%) of participants had low knowledge about COVID-19, 20.4% (95% CI 17.4–23.8%) had negative attitudes towards COVID-19, and 23.1% (95% CI 19.9–26.6%) had the poor adherence to COVID-19 preventive measures.

**Table 2:** COVID-19 epidemic-related factors of the participants

Variables	*n* (%)
Source of information about COVID-19 (multiple response)	
Government media	310 (48.7)
Health workers	230 (36.2)
Social media	169 (26.6)
Friends	98 (15.4)
Family members	76 (11.9)
Infected with COVID-19	
Yes	258 (40.6)
No	378 (59.4)
Family members infected with COVID-19	
Yes	303 (47.6)
No	333 (52.4)
Got COVID-19 vaccine	
Yes	403 (63.4)
No	233 (36.6)

**Table 3:** Level of knowledge about COVID-19, attitudes towards COVID-19 and adherence to COVID-19 preventive measures among the participants

Variables	*n* (%)	95% CI for percent
Knowledge about COVID-19		
High	131 (20.6)	17.5–24.0
Moderate	265 (41.7)	37.8–45.6
Low	240 (37.7)	34.0–41.6
	Mean ± SD: 22.59 ± 4.70, Minimum 9.00, Maximum 33.00	
Attitudes towards COVID-19		
Positive	277 (43.6)	39.7–47.5
Neutral	229 (36.0)	32.3–39.9
Negative	130 (20.4)	17.4–23.8
	Mean ± SD: 38.21 ± 6.14, Minimum 28.00, Maximum 50.00	
Adherence to COVID-19 preventive measures		
Good	250 (39.3)	35.5–43.2
Moderate	239 (37.6)	33.8–41.5
Poor	147 (23.1)	19.9–26.6
	Mean ± SD: 51.78 ± 12.23, Minimum 26.00, Maximum 70.00	

The factors associated with adherence to COVID-19 preventive measures among the participants by the simple and multiple logistic regression analysis are described in Table 4. After adjusting the potential confounders, the factors such as age, ethnicity, occupation, education, monthly family income, and level of knowledge were significantly associated with adherence to COVID-19 preventive measures. Age was a strong predictor of adherence to COVID-19 preventive measures, with significantly increased odds among younger age groups. The odds of having poor adherence to COVID-19 preventive measures were 3.13 times higher in participants aged 31–40 years than those aged elder than 40 years (AOR: 3.13, 95% CI 1.62–6.05). Likewise, the participants aged 30 years and younger were more likely to have poor adherence to COVID-19 preventive measures compared with those who aged elder than 40 years (AOR: 3.22, 95% CI 1.75–5.92). The risk of poor adherence to COVID-19 preventive measures was also increased by 2.52 times in Burmese ethnicity, compared with others (AOR: 2.52, 95% CI 1.44–4.39). The participants with their own businesses were more likely to have poor adherence to COVID-19 preventive measures than the government staff (AOR: 3.19, 95% CI 1.15–8.87). The participants with high school education level and below were 1.64 times more likely to be poor in adherence to COVID-19 preventive measures than those who were with above high school education level (AOR: 1.64, 95% CI 1.02–2.69). The participants earning below 280.90 USD of monthly family income were 51% increase in poor adherence to COVID-19 preventive measures compared with the participants with 280.90 USD and above monthly family income (AOR: 1.51, 95% CI 1.01–2.29). In addition, the odds of having poor adherence to COVID-19 preventive measures was 90% higher in participants with low knowledge about COVID-19, compared with their counterpart (AOR: 1.90, 95% CI 1.26–2.88).

**Table 4:** Factors associated with adherence to COVID-19 preventive measures among the participants

Variables	Poor adherence		COR (95% CI)	*p* value	AOR (95% CI)	*p* value
	No *n* (%)	Yes *n* (%)				
Sex						
Female	267 (79.5)	69 (20.5)	1.00			
Male	222 (74.0)	78 (26.0)	1.36 (0.94–1.97)	0.103		
Age						
> 40	134 (87.0)	20 (13.0)	1.00		1.00	
31–40	130 (76.5)	40 (23.5)	2.06 (1.14–3.71)	0.016	3.13 (1.62–6.05)	0.001
≤ 30	225 (72.1)	87 (27.9)	2.59 (1.52–4.41)	< 0.001	3.22 (1.75–5.92)	< 0.001
Marital status						
Single	179 (77.8)	51 (22.2)	1.00			
Married	273 (77.6)	79 (22.4)	1.02 (0.68–1.51)	0.939		
Separate	12 (85.7)	2 (14.3)	0.59 (0.13–2.69)	0.492		
Divorced	5 (41.7)	7 (58.3)	4.91 (1.49–16.14)	0.009		
Widowed	20 (71.4)	8 (28.6)	1.40 (0.58–3.37)	0.448		
Ethnicity						
Others^†^	116 (85.3)	20 (14.7)	1.00		1.00	
Burmese	373 (74.6)	127 (25.4)	1.98 (1.18–3.31)	0.010	2.52 (1.44–4.39)	0.001
Occupation						
Government staff	61 (91.0)	6 (9.0)	1.00		1.00	
Dependant	124 (77.5)	36 (22.5)	2.95 (1.18–7.38)	0.021	2.72 (0.96–7.69)	0.060
Unskilled laborer	42 (65.6)	22 (34.4)	5.33 (1.99–14.25)	0.001	2.93 (0.93–9.19)	0.066
Own business	107 (75.4)	35 (24.6)	3.33 (1.32–8.36)	0.011	3.19 (1.15–8.87)	0.026
Private employee	155 (76.4)	48 (23.6)	3.15 (1.28–7.74)	0.012	2.37 (0.89–6.32)	0.084
Education^**⁎**^						
> High school education level	203 (83.2)	41 (16.8)	1.00		1.00	
≤ High school education level	286 (73.0)	106 (27.0)	1.84 (1.23–2.75)	0.003	1.64 (1.02–2.69)	0.050
Living situation						
Alone	23 (88.5)	3 (11.5)	1.00			
With family	431 (76.1)	135 (23.9)	2.40 (0.71–8.12)	0.159		
With friends	35 (79.5)	9 (20.5)	1.97 (0.48–8.06)	0.345		
Household member						
≥ 4	261 (77.2)	77 (22.8)	1.00			
< 4	228 (76.5)	70 (23.5)	1.04 (0.72–1.51)	0.832		
Monthly family income (USD)						
≥ 280.90	264 (81.0)	62 (19.0)	1.00		1.00	
< 280.90	225 (72.6)	85 (27.4)	1.61 (1.11–2.34)	0.012	1.51 (1.01–2.29)	0.050
Comorbidity						
Present	76 (86.4)	12 (13.6)	1.00		1.00	
Absent	413 (75.4)	135 (24.6)	2.07 (1.09–3.92)	0.026	1.53 (0.74–3.15)	0.246
Infected with COVID-19						
Yes	216 (83.7)	42 (16.3)	1.00		1.00	
No	273 (72.2)	105 (27.8)	1.98 (1.33–2.95)	0.001	1.55 (0.99–2.43)	0.055
Family members infected with COVID-19						
Yes	240 (79.2)	63 (20.8)	1.00			
No	249 (74.8)	84 (25.2)	1.29 (0.89–1.86)	0.186		
Got COVID-19 vaccine						
Yes	319 (79.2)	84 (20.8)	1.00			
No	170 (73.0)	63 (27.0)	1.41 (0.97–2.05)	0.075		
Low knowledge						
No	316 (79.8)	80 (20.2)	1.00		1.00	
Yes	173 (72.1)	67 (27.9)	1.53 (1.05–2.22)	0.026	1.90 (1.26–2.88)	0.002
Negative attitudes						
No	398 (78.7)	108 (21.3)	1.00		1.00	
Yes	91 (70.0)	39 (30.0)	1.58 (1.03–2.43)	0.038	1.39 (0.87–2.20)	0.164

^†^Ethnicity was categorized as “Others” (Rakhine, Kayin, Chin, Mon, and Shan) and “Burmese”

⁎Education was categorized as “above high school education level” (college and university to graduate and above) and “high school education level and below” (illiterate to high school education)

## Discussion

The COVID-19 pandemic is no longer considered a public health emergency by WHO but the virus is still circulating within populations. In an effort to mitigate the community spread of disease, taking preventive measures for COVID-19 should be continued even after vaccination. This study assessed the level of adherence to COVID-19 preventive measures among the residents in the Yangon Region, Myanmar, beyond the peak of the third wave. The evidences can identify the strategies for improving the adherence to COVID-19 preventive measures among the residents, which may guide the future actions and policymaking during the current pandemic or future pandemics. The majority of participants in this study were aged 30 years or younger and it was in accordance with the findings of a previous Myanmar study [25] and other similar literature assessing adherence to COVID-19 preventive measures [20–23, 28, 38, 39]. Among the participants, most of them (31.9%) were private employees and the previous study done in Myanmar described that most were working outside the home [25]. In similar previous studies, most participants were farmers or pastoralists [22, 38, 39], those who were not working or unemployed [21, 40], governmental employed [28], and jobless or students [23].

In education, one-third of the participant passed the high school education level and it was in keeping with the previous Myanmar study which stated that nearly one-third of the participants had a high school education level [25]. The other studies have shown that most of the participants were primary school level [38, 40], secondary education level [22], undergraduate [30], and university [21]. There were 13.8% of participants who had comorbidities in the current study and the previous studies done in Eastern Ethiopia and Congo also described 12.4% and 13.9% of participants had chronic diseases, respectively [23, 28]. However, another study done in Ethiopia stated that most of the participants, 90.2%, had chronic disease [40]. It seemed possible that the variability of these sociodemographic findings might be due to the differences in geographical and socioeconomic backgrounds, sample size, and categories of variables. The possible explanation for the consistent findings with previous literatures might be due to the similarity of demographic factors, characteristics of participants, source of population, and inclusion criteria.

In this study, 23.1% of the participants had poor adherence to COVID-19 preventive measures and it was lower than the findings of the similar studies carried out in Saudi Arabia, 2021 (44.1%) [21], Thailand, 2022 (45.2%) [41], Congo, 2021 (60.3%) [23], and Ethiopia, 2020–2021 (31.3–91.7%) [20, 22, 28, 38–40, 42–44]. These differences might be due to the distinctions of the study population, geographical background, socioeconomic status, impact of the COVID-19 pandemic in the study areas, and usage of the assessment tools across the studies. In Myanmar, the third wave of the COVID-19 epidemic was a more significant impact on human lives and the economy than the first and second waves, and consequently, increased awareness among people might positively influence the preventive behaviours to avoid the transmission of COVID-19.

In this study, nearly half of participants received the information about COVID-19 from the government media (MOH website and Facebook page, and mass media released from Myanmar Radio and Television broadcasting). The other main sources that the participants received the COVID-19-related information were health workers and social media. MOH has been reporting the daily total tests, confirmed cases, and deaths, sharing updated information on COVID-19 vaccination status occasionally, and providing advice for the public and guidelines related to precautionary measures [12, 13]. In Myanmar, most of the people obtained health information about COVID-19 from social media, MOH sources, and healthcare personnel [25]. An Ethiopia study carried out among communities identified that radio and health workers were the main sources of information about COVID-19 [22].

Among the total, 37.7% of participants had a low level of knowledge and it was higher than the results of studies done in Bhutan, 2022 (2.7%) [18], Cameroon, 2020 (15.8%) [45], and different areas of Ethiopia, 2021 (4.2–29.3%) [28, 38, 46]. However, it was lower than the results of the studies conducted in Southern Ethiopia, 2021 (38.5%) [43], North Shoa Zone of Ethiopia, 2021 (47.1%) [39], and Northwest Ethiopia, 2020 (49.3%) [20]. Regarding the level of attitudes, 20.4% of the participants had negative attitudes and which was higher than the finding of a study conducted in Saudi Arabia, 2020 (18.8%) [34]. Nevertheless, it was lower than the results of the studies done among the communities in Cameroon, 2020 (31.0%) [45], and various areas of Ethiopia, 2020–2021 (29.4–67.8%) [20, 22, 28, 38, 46]. These discrepancies might be due to the heterogeneity of the study population, geographical background, socioeconomic status, impact of the COVID-19 pandemic in the study areas, usage of the assessment tools, and cutoff points for the level of knowledge and attitudes towards COVID-19.

To reduce the community spread, MOH has been providing the risk communication messages and health education facts regarding wearing the face mask, hand hygiene, physical distancing and environmental disinfection on the official web page and social media page [12]. In this study, although most of the residents got the information about COVID-19 from the government media, there were insufficient for high level of knowledge about COVID-19 and good adherence to preventive measures. It might be due to poor community engagement and realization of public health regulations for the adherence to prevention recommendations. Another explanation for this was that unemployment during pandemic was a critical concern for most people and therefore, they might not be aware for the risk of getting disease and could not follow the prevention protocols (e.g., physical distancing) because their livelihood was dependent on going out to work.

Age, ethnicity, occupation, education, monthly family income, and level of knowledge were associated with adherence to COVID-19 preventive measures among the residents. The young people involved more in daily activities or those who were also going outside frequently for their job had a very risky behaviours for COVID-19 infection. However, young people were less likely to follow preventive measures, and it would be challenging for the containment processes of the virus in the future [47]. This study confirmed that the young participants were more likely to have poor adherence to COVID-19 preventive measures than the elders. It could be due to the fact that the young people might have a misconception of disease or believe that they had strong immunity and the disease was resisted by their immunity [48]. Another possible explanation of this finding was that low knowledge about COVID-19 precautionary measures, education level for realizing it as a public health problem, and support and enforcement of family members to follow the directions of MOH might affect the adherence to COVID-19 preventive measures. This result agreed with the findings of the previous studies done in 2021, in which the age of the participants is associated with adherence to COVID-19 preventive measures [22, 42, 49].

In this study, ethnicity was also associated with adherence to COVID-19 preventive measures. Burmese ethnicity was more likely to have poor adherence to COVID-19 preventive measures compared with the others. It might be due to the distribution of the study population in the Yangon Region. According to the study area, Burmese ethnicity (83.5%) was the main population and the minorities were Kayin, Rakhine, Mon, Chin, and Kachin [50]. In an Ethiopia study, there was also a significant difference between two ethnic groups, Oromo and others [22]. In this study, the participants working own businesses were more likely to have poor adherence to COVID-19 preventive measures than the government staff. This result might be explained by the fact that the participants with own businesses might suggest that they were working in their own workplaces and keeping themselves in a safe workplace compared with others who were going outside or working in crowded places. This result was in accord with the previous studies done in 2020–2022 indicating that occupation was a significant associated factor for adherence to COVID-19 preventive measures [22, 23, 41, 51–53]. However, some published studies conducted in 2020–2021 have been unable to demonstrate this association [25, 38, 46].

A small number of previous studies, 2020–2022 reported that a low level of education was associated with non-adherence [22, 23, 34, 41, 53]. A Hungary study, 2021 done in communities approved that the participants with less than high school education increased the odds of non-adherence by 41% compared with those who with college or university [49]. A study carried out in South Ethiopia, 2021 also stated that the participants with primary school education status were 68% less likely to have good adherence to COVID-19 preventive measures than respondents who could not read and write [40]. In accord with the previous studies, the result of the current study indicated that the odds of poor adherence to COVID-19 preventive measures increased with the low level of education. However, there was no significant association between level of education and adherence to COVID-19 preventive measures in an Ethiopia study, 2021 [38]. In this study, the monthly family income was an associated factor of adherence to COVID-19 preventive measures among the residents. An Ethiopia study, 2021 also showed that the populations with low economic status were more likely to have a poor adherence to COVID-19 preventive measure than those who were in high economic status [28].

A high knowledge could attribute to following the recommended directions of precautionary measures [28]. In the current study, the participants who had low knowledge were less likely to adhere to the COVID-19 preventive measures compared with their counterparts. This result was consistent with the findings of the previous studies done in Myanmar, 2020 [25], India, 2021 [30], Sub-Saharan Africa, 2022 [53], and Ethiopia, 2021 [22, 43, 46]. It could be due to the fact that knowledge about COVID-19 transmission, symptoms, severity, and precautionary measures might positively impact on adherence to COVID-19 preventive measures. People with low knowledge might be less likely of adhering to recommended preventive measures and it could be a risk factors for the disease to spread through the community. Nevertheless, some previous studies did not support this finding, reporting that level of knowledge about COVID-19 was not associated with adherence to preventive measures [38, 40].

The exploration of attitudes towards COVID-19 was more critical due to the increasing surge of misinformation in the community which could affect the disease spread. The positive attitudes towards COVID-19 among the population impacted the high level of preventive behavioral practices towards COVID-19 [46, 54]. In some community studies, the significant finding was identified that there was an association between the level of attitudes towards COVID-19 and adherence to preventive measures among the participants [38, 41], however, the finding in the current study did not support these previous literatures. It might be due to the facts that attributed to the attitudes towards COVID-19 such as awareness of population on the disease spread and severity, expansion of vaccination against COVID-19, and risk communication from the main sources of information. It was also in line with the findings of the studies done in the Oromia regional state of Ethiopia and South Ethiopia which reported that attitudes towards COVID-19 preventive measures was not associated with adherence to the prevention of COVID-19 [22, 40].

Married people were more likely to have a good adherence to COVID-19 preventive measures and a significant association of marital status with adherence to COVID-19 preventive measures has been described in some published studies [28, 40, 43, 46]. However, there was no association between marital status of the participants and adherence to COVID-19 preventive measures in this study. Adherence to preventive measures against COVID-19 between the marital status groups were not too different and it might be due to the facts such as psychosocial support and occupy a central position.

This study was a community-based study conducted through the face-to-face interviews with the COVID-19 precautionary measures to assess the real response of adherence to preventive measures against COVID-19, instead of the online survey. In addition, this study was carried out during the highly impacted wave of COVID-19 in Myanmar through governmental mitigation measures. Although there are no restrictions including travel bans, lockdown, and the mandatory use of a face mask currently, retroactive analysis of this study could be applied to not only COVID-19 but also the future pandemics to control the disease spread. The questionnaires used in this study were constructed based on the previous literature and adapted to fit with the study population after checking the reliability test, Cronbach’s α.

However, there were some limitations in this study. First, the study was a cross-sectional design and it might have been difficult to determine the cause–effect relationship between independent variables and the level of adherence to COVID-19 preventive measures. Second, the representativeness of the entire population might not be encountered in this study since the participants were recruited using a convenience sampling method. This limitation could be addressed by recruiting a large sample size of population using random sampling method for the equal chance of being selected. Third, due to the use of multistage non-probability sampling procedure, the risk of wide variations and sampling bias could be occurred in this study and this might lead to a weakness in the analysis without considering for weighting that could be able to deter the accuracy of non-probability sample survey estimates. Fourth, social–desirability bias might be occurred due to the responses of suggested COVID-19 preventive measures that were socially acceptable by the participants, regardless of their poor actual implementation. This limitation could be addressed by providing the indirect questioning, avoiding the leading questions that can influence a participant’s response, and usage of response option to get the more specific answer. Fifth, the validity of the response could not be assessed by the observation checklist in current study. Lastly, the reasons for the adherence status of the residents could not be explained in this study and therefore, deeper insights into the reasons or barriers of adherence should be further explored by the qualitative study.

## Conclusions

There was nearly one-fourth of participants with low level of adherence to COVID-19 preventive measures in this study. Age, ethnicity, occupation, education, monthly family income, and level of knowledge were predictors of adherence to preventive measures against COVID-19. Therefore, risk communication through widely used mainstream media such as government media (MOH website and Facebook page, Myanmar Radio and Television broadcasting), health workers, and other social media should be provided to increase the knowledge and scale up the community’s awareness of adherence to preventive measures against COVID-19. It would have required the implementations of risk communication and community engagement program, and community awareness program to improve an adequate awareness of COVID-19 infection and adherence to preventive measures among the population. In addition, the continuation of public health surveillance and announcement of updated information about COVID-19 to the public, enforcement of directions, regulations, and advice for the public towards COVID-19 preventive measures recommended by the health institution, and revitalization of legal enforcement for the COVID-19 mitigation measures by the government depending on the detection of confirmed cases are crucial to improve the adherence to COVID-19 preventive measures through the community. Implementing the preventive measures during the transmission stage is critical for reducing the likelihood of disease emergence and minimizing the impact of pandemic. Although this study focused on the public awareness and adherence to COVID-19 preventive measure, the findings can contribute to halting the community spread in the future pandemics.

### Supplementary Information

Supplementary Material 1: English version of the questionnaire.

Supplementary Material 2: Myanmar version of the questionnaire.

Supplementary Material 3: Minimal data.

## Data Availability

All relevant data are within the manuscript and its additional files. This is no separate data set to share.

## Abbreviations

AOR: Adjusted odds ratio
CFR: Case fatality rate
CI: Confidence interval
COR: Crude odds ratio
COVID-19: Coronavirus disease 2019
HIV: Human immunodeficiency virus
IQR: Interquartile range
MOH: Ministry of health
MOHS: Ministry of health and sports
SARS-CoV-2: Severe acute respiratory syndrome coronavirus 2
