# Supplementary material for: Adherence to COVID-19 preventive measures among residents in selected townships, Yangon Region, Myanmar: a community-based cross-sectional study
Source: Trop Med Health. 2024 May 11;52:36. doi: 10.1186/s41182-024-00603-6 (PMC11088027; doi:10.1186/s41182-024-00603-6)
Supplement: Supplementary file 1 — Supplementary Material 1: English version of the questionnaire. [file 41182_2024_603_MOESM1_ESM.pdf]

Questionnaires

Code No. ___/___/___

**Sociodemographic characteristics**	
Age: years	Sex: ☐ Male ☐ Female
Address: Township	Monthly family income: Kyats
Marital status:<br>☐ Single<br>☐ Married<br>☐ Separate<br>☐ Divorced<br>☐ Widowed	
Ethnicity:<br>☐ Burmese<br>☐ Kayin<br>☐ Rakhine<br>☐ Shan<br>Others ……………………………	Religious:<br>☐ Buddhist<br>☐ Christian<br>☐ Muslim<br>☐ Hindus<br>Others …………………………………
Occupation:<br>☐ Dependent<br>☐ Unskilled laborer<br>☐ Own business<br>☐ Private employee<br>☐ Government staff	Level of education:<br>☐ Illiterate<br>☐ Read and write<br>☐ Primary school education level<br>☐ Middle school education level<br>☐ High school education level<br>☐ College and university<br>☐ Graduate and above
Living situation:<br>☐ Alone<br>☐ With family<br>☐ With friends<br>☐ Others, please specify ……………………………….	
Family member:	
Comorbid disease: ☐ Yes ☐ No	

COVID-19 Epidemic-related factors		
Sources of information about COVID-19 (multiple response)<br>☐ Government media<br>☐ Social media<br>☐ Health workers<br>☐ Family members<br>☐ Friends<br>Others ………………………………………………		
Infected with COVID-19	☐ Yes	☐ No
Family members infected with COVID-19	☐ Yes	☐ No
Got COVID-19 vaccine	☐ Yes	☐ No

Knowledge about COVID-19

1 COVID-19 is an emerging disease.

☐ Yes ☐ No ☐ Don't know

2 COVID-19 can easily spread from person to person.

☐ Yes ☐ No ☐ Don't know

3 COVID-19 can transmit by

☐ Airborne transmission

☐ Blood transfusion

☐ Droplet transmission

☐ Eating wild animals

☐ Drinking contaminated water

☐ Unsafe sex

☐ Direct contact with infected person

☐ Indirect contact with objects used by the infected person

4 The common symptoms of COVID-19 are

☐ Fever

☐ Vomiting

☐ Diarrhoea

☐ Fatigue

☐ Loss of taste or smell

☐ Dry cough

☐ Yellowish discoloration of skin

5 COVID-19 infected people can be asymptomatic.

☐ Yes ☐ No ☐ Don't know

6 Asymptomatic infected person can transmit the COVID-19 disease.

☐ Yes ☐ No ☐ Don't know

7 COVID-19 disease is more likely to develop severe illness in:

☐ Children

☐ Chronic smokers

☐ People with diabetes mellitus

☐ People with cardiovascular diseases

☐ People with chronic respiratory diseases

8	Development of specific treatment for COVID-19 is still working.			
	☐ Yes	☐ No	☐ Don’t know	
9	Currently, there is a vaccine for COVID-19.			
	☐ Yes	☐ No	☐ Don’t know	
10	COVID-19 vaccination can reduce the risk of hospitalization.			
	☐ Yes	☐ No	☐ Don’t know	
11	COVID-19 vaccination can reduce the risk of serious illness.			
	☐ Yes	☐ No	☐ Don’t know	
12	COVID-19 spread can be prevented by:<br>☐ covering the nose and mouth by bent elbow or tissue while coughing or sneezing<br>☐ Avoiding the crowded places<br>☐ Disinfecting the frequently touched surfaces and objects<br>☐ Staying six feet apart from someone<br>☐ Wearing the face mask when going outside<br>☐ Frequently hand washing with soap and water at least 20 seconds<br>☐ Avoiding the going out unnecessarily			

Attitudes towards COVID-19						
No.	Statements	Answers				
		Strongly Agree	Agree	Uncertain	Disagree	Strongly Disagree
1	COVID-19 is a very important public health problem.	☐	☐	☐	☐	☐
2	Community is responsible for implementing preventive measures of COVID-19	☐	☐	☐	☐	☐
3	People can be fatal if infected by COVID-19.	☐	☐	☐	☐	☐
4	COVID-19 can be prevented.	☐	☐	☐	☐	☐
5	The close contact person should be quarantined immediately.	☐	☐	☐	☐	☐
6	COVID-19 cannot spread from asymptomatic infected person.	☐	☐	☐	☐	☐
7	People with underlying disease are more likely to get severe illness if they are infected.	☐	☐	☐	☐	☐
8	COVID-19 can be prevented by influenza vaccine.	☐	☐	☐	☐	☐
9	Older people should be prioritized group for COVID-19 vaccination.	☐	☐	☐	☐	☐
10	The daily preventive measures should not be required after vaccination.	☐	☐	☐	☐	☐

Adherence of preventive measures for COVID-19						
**Within last 14 days**		**Always**	**Often**	**Sometimes**	**Rarely**	**Never**
1	Avoid going to crowded places.	☐	☐	☐	☐	☐
2	Avoid unnecessary going out or stay at home	☐	☐	☐	☐	☐
3	Keep 6 feet physical distancing	☐	☐	☐	☐	☐
4	Avoid greeting with handshaking, hugging and kissing	☐	☐	☐	☐	☐
5	Frequent hand washing with sanitizer or soap and water at least for 20 seconds	☐	☐	☐	☐	☐
6	Avoid touching eye, nose and mouth with unwashed hand or finger	☐	☐	☐	☐	☐
7	Covering nose and mouth with bent elbow or tissue when coughing or sneezing	☐	☐	☐	☐	☐
8	Wearing the face masks when going outside	☐	☐	☐	☐	☐
9	Cleaning and disinfect frequently touched objects and surfaces	☐	☐	☐	☐	☐
10	Usage of bleach for disinfection of household surfaces	☐	☐	☐	☐	☐
11	Avoid unprotected direct contact with live animals	☐	☐	☐	☐	☐
12	Adequate sleeping	☐	☐	☐	☐	☐
13	Eating nutritious food	☐	☐	☐	☐	☐
14	Taking vitamin or supplements	☐	☐	☐	☐	☐
