# Supplementary material for: Adherence to COVID-19 preventive measures among residents in selected townships, Yangon Region, Myanmar: a community-based cross-sectional study
Source: Trop Med Health. 2024 May 11;52:36. doi: 10.1186/s41182-024-00603-6 (PMC11088027; doi:10.1186/s41182-024-00603-6)
Supplement: Supplementary file 2 — Supplementary Material 2: Myanmar version of the questionnaire. [file 41182_2024_603_MOESM2_ESM.pdf]

မေးခွန်းလွှာ

ကုတ်နံပါတ်. ___/___/___

ကိုယ်ရေးအချက်အလက်များ			
အသက်	နှစ်	လိင် ☐ ကျား	☐ မ
လိပ်စာ	မြို့နယ်	မိသားစု လစဉ်ဝင်ငွေ:	ကျပ်

အိမ်ထောင်ရေး

- ☐ လူပျို/အပျို
- ☐ အိမ်ထောင်သည်
- ☐ အတူမနေ
- ☐ အိမ်ထောင်ကွဲ
- ☐ မုဆိုးဖို/မ

လူမျိုး	ဘာသာ
☐ ဗမာ	☐ ဗုဒ္ဓ
☐ ကရင်	☐ ခရစ်ယာန်
☐ ရခိုင်	☐ မူစလင်
☐ ရှမ်း	☐ ဟိန္ဒူ
အခြား	အခြား ....

အလုပ်အကိုင်	ပညာရေး
☐ မှီခို	☐ စာမတတ်
☐ ကျပန်း	☐ ရေးတတ်ဖတ်တတ်
☐ ကိုယ်ပိုင်လုပ်ငန်း	☐ အခြေခံပညာ
☐ ပုဂ္ဂလိက/ကုမ္ပဏီဝန်ထမ်း	☐ အလယ်တန်း
☐ အစိုးရဝန်ထမ်း	☐ အထက်တန်း
	☐ ကောလိပ်/တက္ကသိုလ်
	☐ ဘွဲ့ရနှင့်အထက်

နေထိုင်မှုပုံစံ

- ☐ တစ်ဦးတည်း
- ☐ မိသားစုနဲ့အတူ
- ☐ သူငယ်ချင်းနှင့်အတူ
- ☐ အခြား ....

အတူနေမိသားစုဦးရေ

ရောဂါအခံရှိခြင်း ☐ ရှိ ☐ မရှိ

<table>
<tr><th colspan="3">COVID-19 ရောဂါကူးစက်မှုဆိုင်ရာအချက်များ</th></tr>
<tr><td colspan="3">COVID-19 ရောဂါနှင့်ပတ်သတ်သော အချက်အလက်များအား သိရှိရသည့်နယ်ပယ်များမှာ
(တစ်ခုထက်ပို၍ဖြေဆိုနိုင်ပါသည်)
☐ နိုင်ငံပိုင် မီဒီယာများ
☐ လူမှုရေးဆိုင်ရာ အသုံးပြုသောမီဒီယာများ
☐ ကျန်းမာရေးဝန်ထမ်းများ
☐ မိသားစုဝင်များ
☐ မိတ်ဆွေသူငယ်ချင်းများ
အခြား ....</td></tr>
<tr><td>မိမိအားကူးစက်ခံရခြင်း</td><td>☐ ရှိ</td><td>☐ မရှိ</td></tr>
<tr><td>မိသားစုဝင်များအားကူးစက်ခံရခြင်း</td><td>☐ ရှိ</td><td>☐ မရှိ</td></tr>
<tr><td>ကာကွယ်ဆေးထိုးနှံထားခြင်း</td><td>☐ ရှိ</td><td>☐ မရှိ</td></tr>
</table>

COVID-19 ရောဂါဆိုင်ရာ ဗဟုသုတမေးခွန်းများ	
၁	COVID-19 ရောဂါသည် အသစ်တွေ့ရှိရသော ရောဂါဖြစ်သည်။<br>☐ ဟုတ်ပါသည် ☐ မဟုတ်ပါ ☐ မသိပါ
၂	COVID-19 ရောဂါသည် တစ်ဦးမှ တစ်ဦးသို့ လွယ်ကူစွာကူးစက်နိုင်သည်။<br>☐ ဟုတ်ပါသည် ☐ မဟုတ်ပါ ☐ မသိပါ
၃	COVID-19 ရောဂါသည် အောက်ပါနည်းလမ်းများဖြင့် ကူးစက်ပြန့်ပွားနိုင်သည်-<br>☐ လေမှတဆင့် ကူးစက်ခြင်း<br>☐ သွေးသွင်းခြင်းမှတဆင့် ကူးစက်ခြင်း<br>☐ အမှုန်အမွှားများမှတဆင့် ကူးစက်ခြင်း<br>☐ တောကောင်သားများ စားသုံးခြင်း<br>☐ မသန့်စင်သောရေ သောက်သုံးမိခြင်း<br>☐ အကာအကွယ်မဲ့ လိင်ဆက်ဆံခြင်း<br>☐ ရောဂါရှိသောသူနှင့် တိုက်ရိုက်ထိတွေ့မိခြင်း<br>☐ ရောဂါရှိသောသူ၏ အသုံးအဆောင်များနှင့် ထိတွေ့မိခြင်း
၄	COVID-19 ရောဂါတွင် အဖြစ်အများဆုံးသောလက္ခဏာများမှာ<br>☐ ဖျားခြင်း<br>☐ အန်ခြင်း<br>☐ ဝမ်းလျှောခြင်း<br>☐ ပင်ပန်းနွမ်းနယ်ခြင်း<br>☐ အနံ့အရသာပျောက်ခြင်း<br>☐ ချောင်းခြောက်ဆိုးခြင်း<br>☐ အသားဝါခြင်း
၅	COVID-19 ရောဂါရှိသောသူသည် ရောဂါလက္ခဏာ မပြနိုင်ပါ။<br>☐ ဟုတ်ပါသည် ☐ မဟုတ်ပါ ☐ မသိပါ
၆	COVID-19 ရောဂါကူးစက်ခံနေရပြီး လက္ခဏာမပြသော်လည်း အခြားသူများအား ရောဂါကူးစက် နိုင်သည်။<br>☐ ဟုတ်ပါသည် ☐ မဟုတ်ပါ ☐ မသိပါ
၇	COVID-19 ရောဂါသည် အောက်ပါတို့တွင် ပိုမိုဆိုးရွားနိုင်သည်။<br>☐ ကလေးများ<br>☐ ဆေးလိပ်သောက်သုံးသူများ<br>☐ ဆီးချိုရောဂါရှိသူများ<br>☐ နှလုံးရောဂါရှိသူများ<br>☐ နာတာရှည်အဆုတ်ရောဂါရှိသူများ

၈	လက်ရှိတွင် COVID-19 ရောဂါအတွက် သီးသန့်ကုသဆေး ကိုရှာဖွေဖော်ထုတ်ဆဲ ဖြစ်သည်။<br>☐ ဟုတ်ပါသည် ☐ မဟုတ်ပါ ☐ မသိပါ
၉	လက်ရှိတွင် COVID-19 ရောဂါအတွက် ကာကွယ်ဆေးပေါ်ထွက်နေပြီဖြစ်ပါသည်။<br>☐ ဟုတ်ပါသည် ☐ မဟုတ်ပါ ☐ မသိပါ
၁၀	COVID-19 ရောဂါ ကာကွယ်ဆေးထိုးထားခြင်းသည် ရောဂါကြောင့်ဆေးရုံတက်ရောက်ရခြင်းကို လျော့ကျစေနိုင်သည်။<br>☐ ဟုတ်ပါသည် ☐ မဟုတ်ပါ ☐ မသိပါ
၁၁	COVID-19 ရောဂါ ကာကွယ်ဆေးထိုးထားခြင်းသည် ရောဂါပိုမိုပြင်းထန်သော အဆင့်သို့ရောက်ရှိခြင်းကို လျော့ကျစေနိုင်သည်။<br>☐ ဟုတ်ပါသည် ☐ မဟုတ်ပါ ☐ မသိပါ
၁၂	COVID-19 ရောဂါကို အောက်ပါအချက်တို့ကို လုပ်ဆောင်ခြင်းဖြင့် ကာကွယ်နိုင်ပါသည်။<br>☐ နှာစေး ချောင်းဆိုးချိန်တွင် ပါးစပ်နှင့်နှာခေါင်းအား တစ်သျှူး (သို့) တံတောင်ဆစ်ဖြင့် ဖုံးအုပ်ထားခြင်း<br>☐ လူထူထပ်သောနေရာများအား ရှောင်ခြင်း<br>☐ ထိတွေ့မှုများသော အရာဝတ္ထုများ၊ မျက်နှာပြင်များအား ပိုးသတ်ခြင်း<br>☐ တစ်ဦးနှင့် တစ်ဦး ၆ ပေ အကွာမှ ပြောဆိုဆက်ဆံခြင်း<br>☐ အပြင်ထွက်သည့်အခါ နှာခေါင်းစည်းတပ်ခြင်း<br>☐ မကြာခဏ လက်အား ဆပ်ပြာနှင့်ရေကို အသုံးပြု၍ မိနစ် ၂၀ ကြာဆေးကြောခြင်း<br>☐ မလိုအပ်ပဲ အပြင်ထွက်ခြင်းမှ ရှောင်ကြဉ်ခြင်း

COVID-19 ရောဂါနှင့်ပတ်သတ်၍ ခံယူချက်ဆိုင်ရာမေးခွန်းများ						
စဉ်	မေးခွန်းများ	Answers				
		လုံးဝသဘောတူပါသည်	သဘောတူပါသည်	မသိပါ	သဘောမတူပါ	လုံးဝသဘောမတူပါ
၁	COVID-19 ရောဂါသည် အလွန်အရေးကြီးသော ပြည်သူ့ကျန်းမာရေးဆိုင်ရာ ပြဿနာတစ်ရပ် ဖြစ်ပါသည်။	☐	☐	☐	☐	☐
၂	COVID-19 ရောဂါကူးစက်ပြန့်ပွားမှုကာကွယ်ခြင်း ဆိုင်ရာ လုပ်ဆောင်ချက်များ အောင်မြင်ရန် လူတိုင်းတွင် တာဝန်ရှိပါသည်။	☐	☐	☐	☐	☐
၃	COVID-19 ရောဂါကူးစက်ခံရပါက အသက်ဆုံးရှုံး နိုင်သည်။	☐	☐	☐	☐	☐
၄	COVID-19 ရောဂါသည် ကူးစက်ခံရခြင်းမှ ကြိုတင်ကာ ကွယ်နိုင်သော ရောဂါဖြစ်သည်။	☐	☐	☐	☐	☐
၅	COVID-19 ရောဂါရှိသူနှင့် ထိတွေ့မှုရှိလျှင် ချက်ချင်း သီးသန့်ခွဲထားသင့်သည်။	☐	☐	☐	☐	☐
၆	လက္ခဏာမပြသော COVID-19 ရောဂါရှိသူမှ တခြား သူများသို့ ရောဂါမကူးစက်နိုင်ပါ။	☐	☐	☐	☐	☐
၇	ရောဂါအခံရှိသူများ COVID-19 ရောဂါကူးစက်ခံရပါက ရောဂါပိုမိုပြင်းထန်စေနိုင်သည်။	☐	☐	☐	☐	☐
၈	ရာသီတုတ်ကွေးအတွက် အသုံးပြုသော ကာကွယ်ဆေး ထိုးခြင်းဖြင့် COVID-19 ရောဂါအား ကာကွယ်နိုင်သည်။	☐	☐	☐	☐	☐
၉	COVID-19 ရောဂါ ကာကွယ်ဆေးထိုးနှံရာတွင် သက်ကြီးရွယ်အိုများအား ဦးစားပေးထိုးနှံသင့်သည်။	☐	☐	☐	☐	☐
၁၀	COVID-19 ရောဂါ ကာကွယ်ဆေးထိုးနှံပြီးနောက် နေ့စဉ်လုပ်ဆောင်နေသော ရောဂါကာကွယ်ရေးလုပ်ငန်းများ ဆက်လက်လုပ်ဆောင်ရန် မလိုအပ်တော့ပါ။	☐	☐	☐	☐	☐

COVID-19 ရောဂါ ကာကွယ်ရေးဆိုင်ရာ လုပ်ဆောင်ချက်များအား လိုက်ပါလုပ်ဆောင်မှု						
လွန်ခဲ့သော ၁၄ ရက်အတွင်း		အမြဲတမ်းလုပ်	မကြာခဏ	တခါတရံ	လုပ်ဆောင်ခဲ့သည်	မလုပ်ဆောင်ပါ
၁	လူထူထပ်သောနေရာများအား သွားရောက်ခြင်းမှ ရှောင်ကြဉ်ခြင်း	☐	☐	☐	☐	☐
၂	မလိုအပ်ပဲ အပြင်ထွက်ခြင်းမှ ရှောင်ကြဉ်ခြင်း (သို့) အိမ်တွင်းသာနေထိုင်ခြင်း	☐	☐	☐	☐	☐
၃	တစ်ဦးနှင့် တစ်ဦး ၆ ပေ အကွာမှ ပြောဆိုဆက်ဆံခြင်း	☐	☐	☐	☐	☐
၄	တစ်ဦးနှင့် တစ်ဦး လက်ဆွဲနှုတ်ဆက်ခြင်း၊ ပွေ့ဖက်ခြင်းနှင့် နမ်းရှုံ့ခြင်းအား မပြုမိစေရန် ရှောင်ကြဉ်ခြင်း	☐	☐	☐	☐	☐
၅	အရက်ပျံပါသော လက်သန့်ဆေးရည် (သို့) ဆပ်ပြာနှင့် ရေတို့ဖြင့် စက္ကန့် ၂၀ ကြာ မကြာခဏလက်ဆေးခြင်း	☐	☐	☐	☐	☐
၆	မျက်စိ၊ နှာခေါင်းနှင့် ပါးစပ်တို့အား မိမိလက်ဖြင့် ထိတွေ့မှုမရှိစေရန် ရှောင်ကြဉ်ခြင်း	☐	☐	☐	☐	☐
၇	နှာစေး ချောင်းဆိုးချိန်တွင် ပါးစပ်နှင့်နှာခေါင်းအား တံတောင်ဆစ် (သို့) တစ်သျှူးဖြင့် ဖုံးအုပ်ထားခြင်း	☐	☐	☐	☐	☐
၈	အပြင်ထွက်သည့်အခါ နှာခေါင်းစည်းတပ်ခြင်း	☐	☐	☐	☐	☐
၉	မကြာခဏ အသုံးပြုလေ့ရှိသော အသုံးအဆောင်များ၊ မျက်နှာပြင်များအား ပိုးသတ်ခြင်း	☐	☐	☐	☐	☐
၁၀	အိမ်ကြမ်းပြင်၊ နံရံများအား ပိုးသတ်ဆေးဖျန်းခြင်း	☐	☐	☐	☐	☐
၁၁	တိရစ္ဆာန်များနှင့် အကာအကွယ်မဲ့ တိုက်ရိုက်ထိတွေ့မှုမှ ရှောင်ကြဉ်ခြင်း	☐	☐	☐	☐	☐
၁၂	လုံလောက်စွာ အိပ်စက်ခြင်း	☐	☐	☐	☐	☐
၁၂	အာဟာရရှိသော အစားအသောက်များကို စားသုံးခြင်း	☐	☐	☐	☐	☐
၁၄	ဖြည့်စွက်အားဆေး သောက်သုံးခြင်း	☐	☐	☐	☐	☐
